# Development and optimisation data of a tissue digestion method for the isolation of orthopaedic wear particles

**DOI:** 10.1016/j.dib.2018.07.060

**Published:** 2018-07-31

**Authors:** J. Patel, S. Lal, S.P. Wilshaw, R.M. Hall, J.L. Tipper

**Affiliations:** a Faculty of Biological Sciences, University of Leeds, UK; b School of Mechanical Engineering, University of Leeds, UK

## Abstract

The data contained within this article relate to several enzymatic tissue digestion experiments which were performed to produce an optimised protocol for the digestion of tissue samples. The digestion experiments involved a total of four different digestion protocols. The first protocol involved digestion with proteinase K, without the use of glycine. The second protocol involved digestion with proteinase K in the presence of glycine. The third protocol consisted of proteinase K digestion in the presence of glycine, with more frequent enzyme replenishment. The final protocol was similar to the third protocol but included a papain digestion stage prior to digestion with proteinase K. The data contained within this article are photographs of tissue samples which were captured at key stages of the four protocols and written descriptions based on visual observation of the tissue samples, which document the appearance of the tissue digests.

## Specifications Table

**Table:** 

Subject area	Biology
More specific subject area	Biomaterials
Type of data	Figures
How data was acquired	Samples were photographed using a digital camera and the appearance of samples was described
Data format	Raw
Experimental factors	Tissue samples from animal cadavers (porcine) were formalin fixed and stored in 70% (v/v) ethanol
Experimental features	Tissue samples were weighed and subjected to one of several different enzymatic digestion protocols and photographed at different timepoints
Data source location	Leeds, United Kingdom
Data accessibility	Data is with this article
Related research article	Patel J, Lal S, Nuss K, Wilshaw SP, Rechenberg B, Hall RM and Tipper JL. Recovery of low volumes of wear debris from rat stifle joint tissues using a novel particle isolation method. Acta Biomater 2018 71:339–350.

## Value of the data

• The data show how different reagents affect the tissue digestion process.
• Researchers may use the image files and descriptions to compare the four different tissue digestion protocols outlined in this article.
• The data in this file may be useful for other researchers to refer to when performing any of the tissue digestion protocols outlined here.
• Researchers may also use the data to compare the digestion protocols outlined here to other digestion protocols in the literature.

## 1 Data

The data relate to the results of each of four different enzymatic tissue digestion protocols, all of which involve proteinase K. The first protocol has been used previously to digest serum proteins [1] but had not been tested in tissue samples. The second digestion protocol was performed in the same way as the first but in the presence of glycine. The third protocol also consisted of a method employing proteinase K in the presence of glycine, but with more frequent enzyme replenishment. The final protocol is similar to the third but includes a papain digestion stage. The final protocol has also been used in formalin-fixed ovine and rat tissues [2]. The data are included within this article as embedded image files of photographs taken of fixed tissue samples before, during or after the application of one of the four enzymatic tissue digestion protocols; in addition, data consisting of embedded text containing a brief description based on visual observation of each tissue sample at given timepoints are included. The data are presented in Table 1.

**Table 1:** Data consisting of the results of four digestion protocols.

## 2 Experimental design, materials, and methods

All tissue digestion procedures were carried out at 50 °C on an orbital shaker. Samples consisted of cadaveric porcine pericapsular stifle joint tissue that was formalin fixed and stored in 70% (v/v) ethanol.

Tissue samples of 1 g (wet weight) were added to plastic tubes and subjected to the digestion procedure described in [1], with or without the addition of 0.33 M glycine at the start of the digestion protocol. Tissue samples were photographed after the digestion process.

Other tissue samples of 0.25 g were prepared and digested as described in [2], without the addition of papain. Instead, proteinase K and sodium dodecyl sulphate (SDS) were immediately added to concentrations of 1 mg ml^−1^ and 0.5% (v/v) respectively. Subsequent proteinase K replenishment was performed in the same way as described in [2], with one further replenishment. Tissue samples were photographed before and after the 48 h digestion process.

Other tissue samples of 0.25 g were prepared and digested exactly as described in [2].
